# Supplementary material for: Assessing ventilation through ambient carbon dioxide concentrations across multiple healthcare levels in Ghana
Source: PLOS Glob Public Health. 2024 Aug 14;4(8):e0003287. doi: 10.1371/journal.pgph.0003287 (PMC11324151; doi:10.1371/journal.pgph.0003287)
Supplement: Attachment — Submitted filename: Response to the Reviewers Comments 6 May 2024.docx [file pgph.0003287.s002.docx]

**Response to the Reviewer’s Comments (***Responses and Recommendations from John Dibato***).**

We wish to thank the reviewers for their thoughtful assessment of our research study, titled “Assessing ventilation through ambient carbon dioxide concentrations across multiple healthcare levels in Ghana”. The authors have revised the manuscript according to the points raised by the reviewers, and our responses are listed below to each proposed point.

| **Reviewer 1** | **Response 1** |
| --- | --- |
| There are a few issues that need to be clarified in the paper.  **1.** It is stated that purposive sampling was used. It would be better if an explanation is provided to indicate why this method of sampling was used. Overall, the justifications used for the type of sampling used. What prompted the selection of the specific study locations in the three municipalities as identified in the study?  **2.** The parameters for the logistic regressions and discussions of the logistics results are missing. It is mentioned that this was carried out, but this has not been indicated anywhere in the paper. In line 213-214 there is a discussion that may allude to these results but from the tables shared it is not possible to locate table one.  **3.** The use of descriptive statistics as the only statistical analysis may not be adequate to validate the conclusions arrived at based on the data and the overall research question of the paper. Consider adjusting the statistical approach used. | **1.** Convenience sampling led to the selection of the three municipalities as the locus for assessment, given the resource constraints on the researchers within the 3-month period of data collection. Next, purposive sampling was used to select a heterogenous distribution of primary, secondary, and tertiary healthcare facilities that were 1) accessible to researchers within a 1 hour travel radius from the metropolitan center of the municipality and 2) actively seeing patients on the day of the visit, so that data taken would more accurately approximate the average user experience during daytime operations. These locations were meant to provide an indicative sample of healthcare facilities in the three municipalities to illustrate the range of built healthcare environments found in Ghana, a resource-limited context.  **2.** We thank the reviewer for pointing out this very important aspect of the analysis. We have now incorporated the logistic regression models into the revised paper. This can be reflected in both the statistical analysis and results sections.  **3.** We thank the reviewer for pointing out this very important aspect of the analysis. As discussed in (2), we have now included a multivariate binomial logistic regression with CO_2_ levels (low vs high) as the outcome and ventilation vs non-ventilation as the main exposure. The model was adjusted for facility level and number of people. Details can be found in the statistical analysis and Results section. |

| **Reviewer 2** | **Response 2** |
| --- | --- |
| I consider the evaluation of ventilation systems in healthcare facilities and hospitals to be of utmost importance, and I commend the authors for their diligent research and assessment. The methodology employed in this study is robust, and the findings offer valuable insights and recommendations. | We thank the reviewer for their time spent evaluating the manuscript and have no additional changes resulting from their response. |
